# Physical work environment and burnout among primary care physicians in Israel: a cross-sectional study

**DOI:** 10.1186/s12875-024-02310-x

**Published:** 2024-02-28

**Authors:** Yaara Bentulila, Liat Lev Shalem, Bar Cohen, Limor Adler

**Affiliations:** 1 Health Division, Maccabi HealthCare Services, Tel Aviv-Jaffa, Israel; 2 Faculty of Medicine, Department of Family Medicine, Tel Aviv University, Tel Aviv, Israel

**Keywords:** Burnout, Prevention, Physical work environment, Primary care

## Abstract

**Background:**

Physician burnout remains a prevalent issue globally, negatively affecting work satisfaction and patient care. However, exploration of the physical work environments of physicians, a potential influencing factor for burnout, remains scarce. The physical work environment is everything that surrounds the physician, including the doctor’s office, the clinic, the clinic’s building, the waiting, and staff rooms. The aims of this study were to describe aspects of the physical work environment of primary care physicians (PCPs) and to explore the association between the physical work environment and burnout.

**Methods:**

In this cross-sectional study, we emailed questionnaires to an online community of PCPs in Israel in October 2021. We asked physicians about their satisfaction with their physical work environment, evaluated elements of the work environment, and assessed burnout status (with the Shirom-Melamed Burnout Measure, SMBM). We used the Chi-square and Mann-Witney tests to compare categorical and continuous variables and used logistic regression for the final model.

**Results:**

Two hundred twenty-one PCPs answered the questionnaire (27.6% response rate). Over a third (35.7%) of respondents reported high burnout. PCPs who were satisfied with their general physical environment had lower burnout rates than those who were unsatisfied (28.1% vs. 47.8%, *p*-value < 0.001). We found positive correlations between general satisfaction with the physical work environment and the scores achieved for the doctor’s office, the clinic, the clinic’s building, and the waiting room. In the multivariate analysis, high satisfaction with the general physical work environment was associated with decreased odds for burnout (OR-0.50, 95% CI 0.25–0.99, *p*-value-0.048).

**Conclusion:**

The doctor’s office, the clinic, the clinic’s building, and the waiting room affected general satisfaction from the physical work environment. High satisfaction with the physical work environment reduced burnout rates. Future studies are needed to determine whether PCPs and managers should invest in the physical work environment to decrease burnout and increase satisfaction.

**Supplementary Information:**

The online version contains supplementary material available at 10.1186/s12875-024-02310-x.

## Background

The phenomenon of burnout among physicians in healthcare systems is extensive and influences both work satisfaction and patient care [1]. Maslach defined burnout as a syndrome consisting of three dimensions: the energetic (emotional and physical exhaustion), the interpersonal (depersonalization/cynicism), and the evaluation (devaluation of personal achievement) [2]. Shirom later defined burnout in work organizations as a chronic negative emotion resulting from dwindling energetic resources due to long-term exposure to stress in and out of the workplace [3]. Healthcare workers (HCWs) have been shown to experience burnout more than workers in other professions (38% vs. 27% in the general population) [4]. Primary care physicians (PCPs) are at an increased risk for burnout [5, 6], with a dramatic increase in burnout since the outbreak of the COVID-19 pandemic [7, 8].

**Fig. 1:**
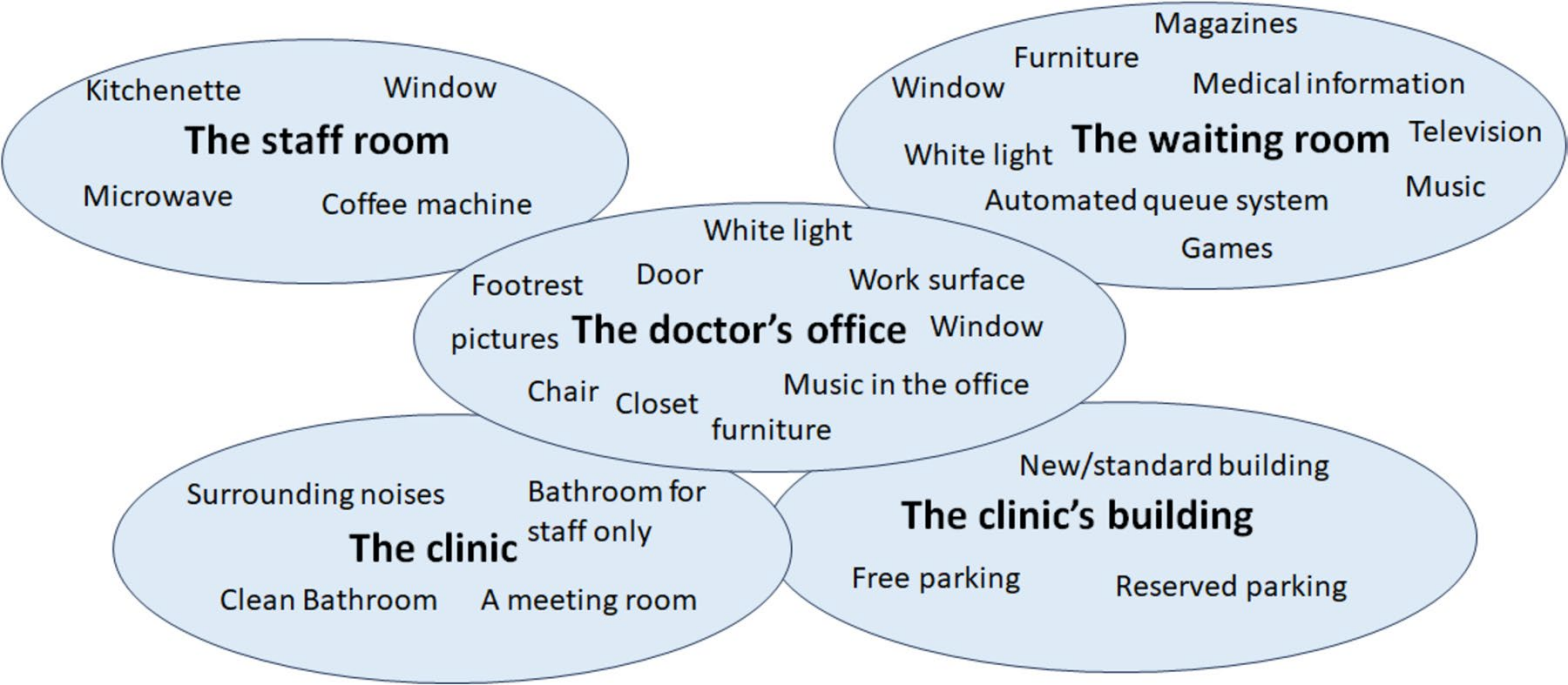
The elements composing the physical work environment of the primary care physician Legend: These are all the specific elements of the physical work environment that we assessed in the study, grouped into five categories: the doctor's office, the clinic, the clinic's building, and the staff and waiting rooms

The physical work environment includes everything that surrounds the worker [9]; in the context of primary care, it consists of the doctor's office, the clinic, the clinic's building, the staff, and waiting rooms (Fig. 1). Studies regarding the physical work environments of HCWs in community settings are scarce. Most of the existing research focuses on the physical work environment in hospitals and specifically on the physical environment of patients [10, 11]. The physical work environment in PCP clinics has hardly been studied.

In Israel, two comprehensive surveys about burnout among HCWs indicated that physicians are the sector that reported the highest burnout rates, with more than 40% of all physicians experiencing burnout [12, 13] (More information about the healthcare system in Israel is provided in Table 1). Many factors were reported in these reports as associated with burnout, of which those most significant were difficulty maintaining work-life balance, high workload, and a physical work environment that burdens the work. While these reports indicate an association between the physical environment and burnout, a comprehensive understanding of this relationship is currently lacking. The specific characteristics of the environment in the hospitals and clinics where the surveys took place, as well as their interactions, were not reported.

**Table 1:** The work pattern of primary care physicians in Israel

The Israeli healthcare system stipulates that all citizens are to have a regular PCP, which they themselves can choose. PCPs can work in one or more HMOs and be paid a regular salary or provide a fee for service. Salary-paid PCPs typically practice in HMO clinics, and PCPs who are paid by fee typically practice in their private clinics or special physicians' offices or special physicians' offices	

Demerouti et al. hypothesize in their model, the Job Demands-Resources (JD-R) model, that burnout is influenced by both job demands (physical workload, physical environment, time pressure, etc.) and job resources (feedback, rewards, job control, etc.) [14]. They suggested that when the external environment lacks resources, the worker struggles to cope with high demands (such as a high workload) and tends to experience burnout. Their work defines the physical environment as the workplace's climate, light, noise, design, and materials.

According to Herzberg's two-factor theory (the motivation-hygiene theory), there are "motivating" factors (internal factors) that contribute to job satisfaction, like achievement, responsibility, recognition, personal growth, and the work itself [15, 16]. Lack of these factors does not necessarily cause dissatisfaction. "Hygiene" factors (external factors) could cause job dissatisfaction. These include salary, organizational policy, administrative structure, relations with others, job security and physical working conditions.

Both models suggest that the physical work environment, whether a job demand or a hygiene factor, influences job dissatisfaction and burnout. As such, evaluating the physical work environment and its impact on PCPs is significant.

This study aimed to describe various aspects of the physical work environment of PCPs in Israel and to explore the association between specific aspects of the physical work environment with satisfaction (from the physical work environment) and the association between satisfaction and burnout. Our initial hypothesis was that when physicians are more satisfied with their physical work environment, they will experience less burnout. We assumed that different components of the physical environment would have a different impact on their satisfaction, with the doctor's office being the most important aspect of the physical environment, as this is the physician's immediate surroundings.

## Methods

### Setting and Study design

We conducted a descriptive cross-sectional study among PCPs working in Israel's healthcare maintenance organizations (HMOs). In 2021, we sent an online questionnaire via text messages and e-mails to 800 PCPs. We used a convenience sample and sent the questionnaire to participants from an online community of PCPs. Emails were sent to a Google group, and text messages were sent through WhatsApp groups. The researchers sent the messages. In the body of the message, there was a link to a Google form. Google Forms is a free platform that allows complete anonymity of the respondents. We asked the physicians about their satisfaction with their physical work environment and included questions reflecting burnout status. The study was approved by the Institutional Review Board (IRB) of Maccabi Healthcare Services (0138–20-MHS). Informed consent was granted by submission of a completed questionnaire.

### The questionnaire

As similar studies exploring the association between burnout and physical work environment among physicians are few, and we did not find any similar study that evaluated it among PCPs, we composed a new questionnaire to fit our goals. The questionnaire (including the satisfaction and physical work environment components) was constructed by a joint discussion between the researchers until an agreement was reached. We tested the feasibility and did a face validity process for the physical work environment component and the satisfaction component of the questionnaire with 10 PCPs. This process supported the suitability of the questionnaire with no significant comments from the participants. For the burnout measure, we used a validated scale, the Shirom-Melamed Burnout Measure (SMBM). An English translation is available in the Supplementary material. The questions were categorized into four subjects:

1. General satisfaction with the physical work environment– with a Likert scale of 1 (not satisfied at all) to 5 (highly satisfied). We converted this variable to a dichotomous variable, satisfied (4–5 on the Likert scale) or dissatisfied (1–3 on the Likert scale).
2. Specific questions about the physical work environment
  - The doctor's office [9–13]—refers to windows, lighting, and additional amenities (footrest, etc.).
  - The assessment of the clinic’s physical infrastructure (henceforth referred to as *the clinic* questions 4.3–4.5, 5)—refers to the existence of a meeting room, staff bathroom, and surrounding noises.
  - The overall assessment of the clinic's building (henceforth referred to as *the clinic’s building*; questions 3, 4.1–4.2) – refers to the nature of the building itself and parking possibilities
  - The staff room (questions 14.1–14.6) – refers to whether a staff room exists in the clinic and whether it is equipped with equipment and supplies to prepare food and beverages.
  - The waiting room (questions 6–8, 8.1–8.6) -refers to windows, lighting, queuing system, entertainment for waiting patients, and furniture.

The clinic is a component that represents the allocation of resources and inner workings of the clinic. The clinic’s building refers to the amenities and condition of the building– whether standard/new or old and parking availability. While for some, there is an overlap between these two components, it is often not the case. Where factors relevant to the clinic's building may be out of the HMOs’ or management’s hands, the clinic itself can often be adjusted and changed. As for the additional components – waiting rooms are meant mostly for patients, but they affect physicians' work by proxy; the staff rooms, where they exist, are a separate space for breaks and socialization among the clinic’s staff.

(3) Burnout questionnaire—we asked PCPs to answer the SMBM. The SMBM is a validated questionnaire to assess burnout [17, 18]. High burnout was considered to be an average score higher than 4. Different cut-off points exist in various studies, above 3 or 4 for burnout and above 4.4 for severe burnout [19–22]. The last report of the Ministry of Health in Israel chose a score of 4, which is the cut-off score we selected for this study [12]. The SMBM has three dimensions of burnout: physical fatigue (questions 1–6, for example, " I feel tired"), cognitive weariness (questions 7–11, for example, "I have difficulty thinking about complex things"), and emotional exhaustion (questions 12–14, for example, " I feel I am not capable of being sympathetic to my patients or coworkers").
(4) Demographic information, including age, gender, employment status, and seniority.

It is worth noting that this study used symptoms of burnout as signifiers of burnout while assuming that the level of symptoms correlates with the level of burnout experienced. We use the term “burnout” in place of “symptoms of burnout” for this purpose; however, the use of this indirect expression should be considered in the context of the results and study design.

Participation in the study was voluntary. PCPs were assured that their responses would remain confidential. Consent to participate was granted by submission of the completed questionnaire. The questionnaire was in Hebrew.

### Sample size

In order to calculate the sample size required for this study, we assumed that the ratio between physicians with and without burnout would be (2:3), as seen in previous reports published by the MoH in Israel [12, 13]. We also assumed that 45% of physicians without symptoms of burnout would be satisfied with their physical work environment compared to 25% of physicians with symptoms of burnout. The sample size required for this aim, with a power of 80% and a significance level of 5%, is 188 (75 and 113 participants, respectively). We sent the questionnaire to a sample of 800 physicians to achieve this goal, assuming a response rate of at least 25%. The response rate within primary care surveys ranges from 10 to 61% [23]. The overall physician response rate to web-based surveys is 35% and varies between different specialties [24]. From our experience, physicians in Israel have a lower response rate to web-based surveys, hence our initial hypothesis of a response rate of around 25%.

### Statistical analysis

Descriptive statistics were used to describe the results. To check for the association between burnout measures (high (i.e., SMBM score > 4) vs. low) and satisfaction from the physical environment (recoded into a dichotomous variable; 4–5 (satisfied) vs. 1–3 (dissatisfied)), we used the Chi-Square test.

We created five continuous variables that represent five components of the physical work environment (the clinic's building, the clinic, the doctor's office, the staff, and waiting rooms; scores were calculated based on the number of items reported for each component (a higher score represents more items in each component, and a better physical work environment, [Fig. 1]) (the scoring index is elaborated in the questionnaire). We used the Mann–Whitney U test to examine the association between these five workspaces and general satisfaction (satisfied vs. dissatisfied).

We used logistic regression to evaluate how multiple variables affect burnout, including general satisfaction from the physical work environment, age (30–44, 45–59, 60 +), gender, specialty status (specialist vs. resident), workplace (HMO clinic vs. other), average weekly working hours (0–19, 20–39, 40 +). The Statistical Package for Social Sciences (SPSS) software version 28 was used for data analysis. For sample size calculations, we used WinPepi version 11.65.

## Results

During October 2021, 221 PCPs answered the questionnaire (27.6% response rate), 61.8% were females, the mean age was 48.2 (SD = 11.1) (range 30–74), and on average, working 32.5 (SD = 24) (range 4–60) hours per week. There were no missing data since all fields in the questionnaire were obligatory.

### Satisfaction from the physical environment

#### Descriptive analysis

*Burnout scores* – the mean SMBM score of PCPs in our study was 3.7 (SD = 0.8), with 35.7% of respondents reporting high burnout (SMBM > 4). In the three dimensions of the SMBM (physical fatigue, emotional exhaustion, and cognitive weariness), 103 (47.2%), 56 (27.1%), and 34 (16.4%) had a high score (> 4), respectively. More females and more PCPs who worked in an HMO branch had high burnout scores (70.9% vs. 56.5%, p-value 0.042 and 82.3% vs. 56.8%, respectively) (Table 2).

**Table 2:** Characteristics of primary care physicians with low and high burnout scores in the Shirom-Melamed Burnout Measure

	Low burnout score (< 4) n (%)	High burnout score (≥ 4) n (%)	*p*-value
**Gender**			
Female	78 (56.5%)	56 (70.9%)	0.042
**Age**			
30–44	56 (45.5%)	*N* = 30, 46.2%	
45–59	38 (30.9%)	*N* = 27, 41.5%	0.132
60 +	*N* = 29, 23.6%	*N* = 8, 12.3%	
**Status**			
Attending	94 (67.7%)	*N* = 61, 77.2%	0.162
Resident	45 (32.4%)	*N* = 18, 22.8%	
**Weekly Working hours**			0.183
0–20	*N* = 17, 13.1%	*N* = 6, 8%	
20–40	*N* = 76, 58.5%	*N* = 39, 52%	
40 +	*N* = 37, 28.5%	*N* = 30, 40%	
**Workplace**			
HMO branch	79 (56.8%)	65 (82.3%)	< 0.001
Other	*N* = 60, 43.2%	*N* = 14, 17.7%	

*General satisfaction* – 128/221 (57.9%) report they were generally satisfied or highly satisfied with their physical work environment (4–5 on the Likert scale).

Characteristics of the physical work environment are presented in Table 3. Some characteristics are worth mentioning: only 18% reported they had reserved parking for PCPs, less than a third (32.7%) reported a designated room for meetings, and only 13.2% reported a white room in the doctor's office.

**Table 3:** Characteristics of the physical work environments

	Affirmative
**Doctor’s office**	
Adjustable chair	201 (93.5%)
A closet	192 (89.3%)
Furniture is standard/new^a^	189 (85.5%)
Clean surface to work on	181 (85%)
A free-opening door	178 (84%)
Pictures on the wall	164 (76.3%)
A window that can be opened	161 (75.6%)
Footrest	73 (34.3%)
White light	28 (13.2%)
Music in the office	9 (4.2%)
**The clinic**	
No surrounding noises / some background noises	174 (78.7%)
Bathrooms are clean and suitable	165 (79.7%)
Bathrooms reserved for staff	115 (54.2%)
Designated room for meeting	70 (32.7%)
**The clinic’s building**	
Free Parking	161 (78.5%)
Standard/new building^a^	167 (75.6%)
Reserved parking for PCPs	38 (18%)
**The staff room**	
A kitchenette with groceries	157 (75.5%)
A microwave	161 (75.2%)
A staff room exists	146 (68.5%)
The staff room is on the same floor as the doctor's office	126 (60.9%)
A window	77 (36.3%)
A coffee machine	49 (23%)
**The waiting room**	
A window that cannot be opened	218 (100%)
Furniture is standard/new	195 (89.8%)
White light	173 (80.5%)
Automated queue system	140 (66%)
Medical information is available for patients	116 (54.7)
Games for children	94 (43.5%)
Television	78 (36.8)
Magazines	66 (31.1)
Music	27 (12.7)

^a^A subjective measure, open to the interpretation of the respondent

#### Univariate and bivariate analysis

PCPs who were satisfied or highly satisfied with their general physical environment had lower rates of burnout (28.1% vs.47.8%, *p*-value < 0.001 in satisfied vs. non-satisfied PCPs, respectively). This trend persisted with all three dimensions of burnout to varying degrees (for physical fatigue – 40.6% vs. 56.7%, *p*-value = 0.027; for cognitive weariness – 8.1% vs. 28.6%, *p*-value < 0.001; for emotional exhaustion – 19.5% vs. 38.1%, *p*-value = 0.004).

**Fig. 2:**
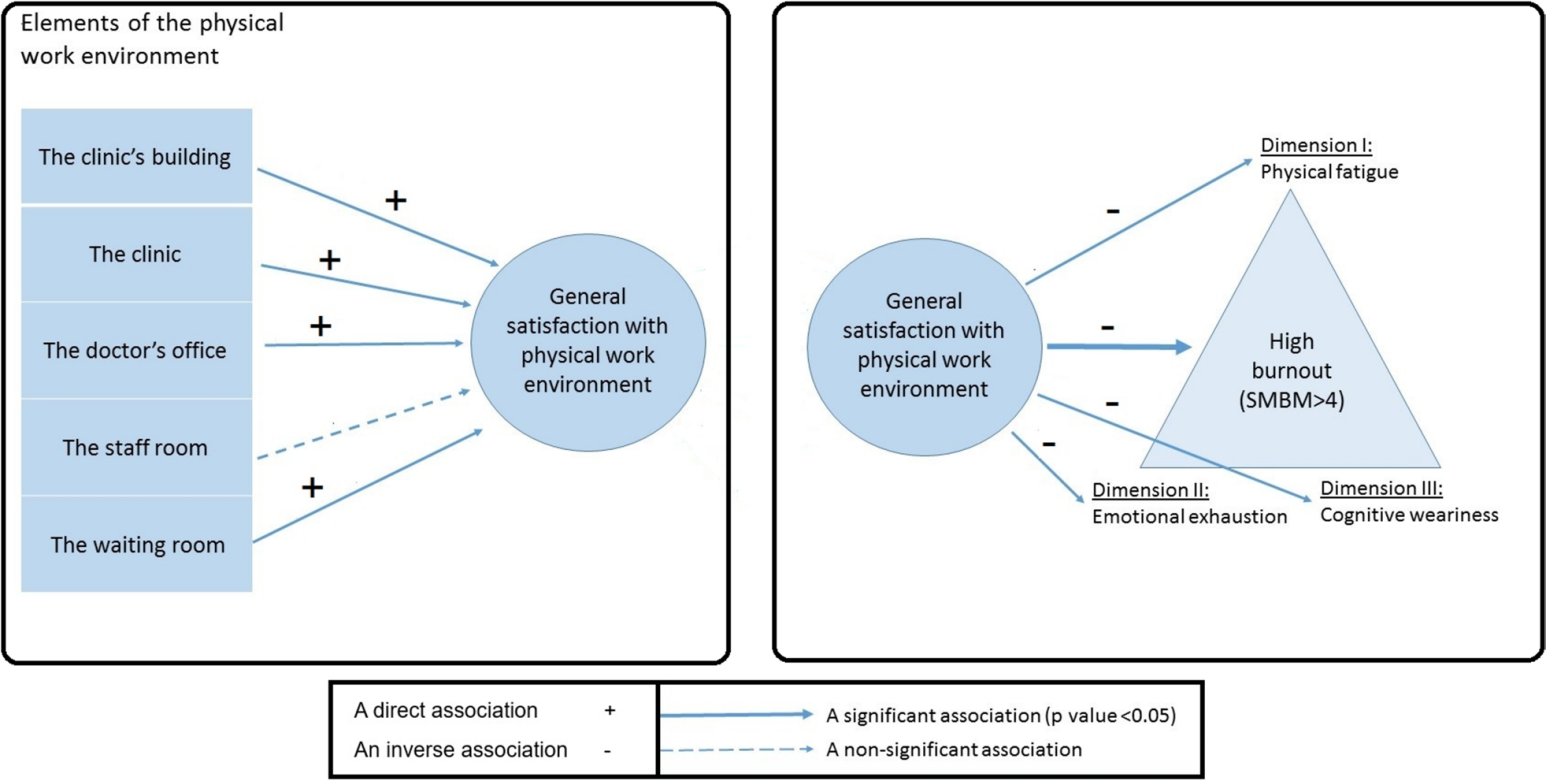
Association between aspects of the work environment, general satisfaction from the work environment, and burnout in primary care physicians. Legend: We found associations between general satisfaction with the physical work environment and the scores achieved for the doctor’s office, the clinic, the clinic's building, and the waiting room. The association was not significant for the staff room. In addition, PCPs who were satisfied or highly satisfied with their general physical environment had lower rates of burnout. This trend persisted with all three dimensions of burnout to varying degrees

When examining the relationship between different aspects of the physical work environment and general satisfaction (high vs. low), we report a positive correlation between general satisfaction with the physical work environment and the scores achieved for the doctor’s office (7.5 vs. 6.6, *p* value < 0.001), the clinic (2.6 vs. 2.1, *p* value = 0.001), the clinic's building (1.8 vs. 1.4, *p* value < 0.001), and the waiting room (4.2 vs. 3.7, *p* value = 0.011). We did not find a significant correlation between the staff room and general satisfaction (3.2 vs. 3.3, *p* value = 0.888) (see Fig. 2). All specific elements of the physical environment are outlined in Table 3.

#### Multivariate analysis

We found that high satisfaction from the physical work environment was associated with low burnout (OR-0.50, 95% CI 0.25–0.99, *p*-value = 0.048). Working in an HMO branch (compared to an independent clinic or a compound with multiple clinics) was associated with high burnout scores (OR-2.46, 95% CI 1.14–5.32, *p*-value 0.022). Age and gender were not associated with burnout. Being a specialist (vs. a resident) and number of working hours did not enter the final model (Table 4).

**Table 4:** Multivariate analysis for high burnout in the SMBM

	OR (95% CI)	*p*-value
High Satisfaction with the general physical work environment	0.50 (0.25–0.99)	0.048
Female Gender	1.85 (0.90–3.82)	0.093
Age		
30–44	Reference	
45–59	1.63 (0.80–3.34)	0.178
60 or above	0.70 (0.25 – 1.97)	0.504
Workplace (working in an HMO clinic)	2.46 (1.14 – 5.32)	0.022

The first step of the model with the variable *satisfaction from the physical work environment* had a Nagelkerke R square of 5.4%. The Nagelkerke R square of the model in the second step, when *age* and *gender* were added, was 11.1%. When the workplace was added, the Nagelkerke R square of the final model was 15.1% (15% of the burnout symptoms could be attributed to variables in the model).

## Discussion

### Main findings

In this study, we report a rate of burnout (SMBM > 4) of 37.5% among respondents. PCPs who were satisfied or highly satisfied with their general physical work environment had a lower prevalence of burnout. We found a positive correlation between general satisfaction from the physical work environment and factors related to the clinic's building, the clinic, the doctor's office, and the waiting room.

### Interpretation

The rate of burnout in our study is 37.5%. The rates of burnout among PCPs, in a systematic review and meta-analysis, were 37% for emotional exhaustion, 28% for depersonalization, and 26% for personal exhaustion [25]. Maslach's theory defined these three dimensions. However, we used the SMBM, which suggests similar but not identical dimensions, including physical fatigue, emotional exhaustion, and cognitive weariness, with rates of 47.2%, 27.1%, and 16.4%, respectively.

Our study found an association between burnout (and all three dimensions of it) and general satisfaction from the physical work environment. This aligns with Rabatin et al.'s study, which found that burned-out clinicians generally report less job satisfaction [26]. Two large studies assessing the work environment's influence on burnout focused on workload, work-life balance, job autonomy, and organizational support [27, 28]. Still, the physical work environment was overlooked. Our study demonstrated that physical conditions must be considered when evaluating the workplace.

We found an association between different aspects of the physical work environment and general satisfaction with the physical work environment. This association was found for the doctor's office, the clinic, the clinic's building, and the waiting room. This is in line with Ulrich's study from 1984, which examined the effects of hospital design on patients and staff. He reported that a window overlooking nature is associated with higher staff satisfaction. Positive results reported by staff were associated with more energy, enthusiasm, and satisfaction with their workplace [10]. A study conducted by Mroczek in 2005 dealt with hospital workers’ perception of their physical work environment [11]. Natural lighting, live music in the hospital hall, good ventilation, water elements, and homey patient rooms were all rated as most significant.

The JD-R model defined the physical environment as a job demand that causes exhaustion [14]. Parts of the physical environment are the climate, light, noise, design, and material of the workplace [9]. These are all aspects of the physical environment we evaluated, including the light in the doctor's office and waiting room, the furniture, windows, surrounding noises, parking, etc. (Fig. 1). Regarding light, several studies demonstrate that increased daylight exposure increases productivity and sleep quality, and reduced visual comfort reduces productivity [29]. Satisfaction with the looks and aesthetics of the office also increases productivity [29].

### Strengths and limitations

This study has several limitations. Firstly, a response rate of 27.6% is less than the average of 35% in web-based surveys among physicians [24]; while this is explained by the cross-sectional and voluntary nature of the study, it should be considered, and a selection bias might exist. Secondly, we used a convenience sample, and as such, it may not represent the entire population of interest. Thirdly, we cannot compare to non-responders, so we cannot evaluate the character of selection bias and its direction. Finally, due to the scarcity of relevant research, we could not use a validated questionnaire regarding the physical work environment; therefore, we composed a questionnaire independently and encouraged further use and validation of it. However, we used a validated questionnaire, the SMBM, to evaluate burnout status.

### Implications

Burnout among physicians affects patients’ health, costs, and physicians' health [30–33]. On a personal level, physicians who are burned out feel more tired, exhausted, inattentive, and irritable [30] and even tend to have more motor vehicle accidents [34]. It can also increase stress and depression among physicians [35]. The professional consequences include a tendency for medical errors, and in severe cases, physicians even leave the organization they work in [36, 37]. More professional outcomes are a risk for malpractice, reduced patient satisfaction, and outcomes [38]. This research yielded several results that may be directly applicable at the individual and organizational levels. Burnout rates were relatively high in this sample, with over a third of respondents reporting symptoms of burnout. This might be due to timing (COVID-19 and its effects on primary care) [39, 40], a possible selection bias, or a combination of both. Interestingly, age and gender did not significantly impact burnout in this study, unlike reported results in past research [41]. Working in a branch of an HMO also correlates with high burnout rates, probably due to the stress in this specific environment.

The question that arises from our findings is whether changes in the physical work environment of PCPs can impact burnout rates like other proven interventions to decrease burnout in physicians [42, 43]. Linzer et al. showed that improving work conditions can reduce burnout rates and increase the satisfaction of PCPs [44]. However, their study focused on communication, workflow, and targeted quality improvement, not the physical work environment.

In light of our findings, we suggest managers of clinics and regional managers of healthcare organizations pay special consideration to the physical work environment of PCPs. This includes a window, new and comfortable furniture in the doctor's office, a chair with a footrest, white light, and pictures. The clinic should have a meeting room, designated personnel, clean bathrooms, and no (or little) surrounding noises. The clinic building should be new or standard and have available parking for physicians. The waiting room should have standard/new furniture, white light, windows, music or television, games for children, and medical information available for patients.

## Conclusion

The doctor's office, the clinic, the clinic's building, and the waiting room affected general satisfaction from the physical work environment. High satisfaction with the physical work environment reduced burnout rates. Future studies are needed to determine whether PCPs and managers should invest in the physical work environment to decrease burnout and increase satisfaction.

### Supplementary Information

**Supplementary Material 1.**

## Data Availability

The data that support the findings of this study are available on request from the corresponding author, LA. The data are not publicly available due to IRB's restrictions.

## Abbreviations

HCW: Healthcare workers
HMO: Healthcare maintenance organizations
JD-R model: Job Demands-Resources model
PCPs: Primary care physicians
SD: Standard deviation
SMBM: Shirom-Melamed Burnout Measure
