# Supplementary material for: Physical work environment and burnout among primary care physicians in Israel: a cross-sectional study
Source: BMC Prim Care. 2024 Feb 28;25:74. doi: 10.1186/s12875-024-02310-x (PMC10900697; doi:10.1186/s12875-024-02310-x)
Supplement: Supplementary file 1 — Supplementary Material 1. [file 12875_2024_2310_MOESM1_ESM.docx]

Dear physician,

Following is a questionnaire regarding the physical work environment in your clinic, and regarding the correlation between physical work environment and burnout.

Physical work environment is a specific aspect of overall work environment, which affects the worker’s senses.

Most of us spend at least half of our time in our work environment.

Physical work environment has an effect on cognitive functioning, mental state, concentration abilities and indirectly on our overall function. This subject was studied extensively in business. When the physical environment is suitable, there is an increase in workers’ satisfaction, functioning and even health. In the healthcare system, this subject has not been thoroughly studied and was mostly focused on hospitals and aspects relevant to patients.

In this questionnaire, we would like to query as to components of physical work environment in your primary clinic, and as to matters concerning personal wellbeing and burnout.

This study will contribute to a better understanding of physical work environment in primary clinics.

The data collected in this study are confidential. No identifying details will be reported at any point during this study. Answers will only be used for research purposes and will be saved completely confidentially.

Your participation in this study is voluntary. By answering the questionnaire, you are expressing consent to participate in the study.

Estimated time to complete this questionnaire is 5 to 10 minutes.

Thank you in advance for your participation!

Dr. Ya’ara Bentoulilah, Dr. Limor Adler

Maccabi Health Services (MHS)

1. **Opening questions**

| Possible answers | Question |
| --- | --- |
| Yes  No | Do you consent to participate in this study? |
|  | Please choose your primary workplace (clinic) and refer to it when answering the following questions |

**Following are several questions regarding various components in the physical work environment in your clinic (refer to your primary workplace) and their characteristics. The questions concern different spaces in the clinic, as follows:**

**2. What is your level of satisfaction with the physical work environment in the clinic (by the physical work environment, we are referring to furniture/design, noise, lighting, ambiance, and spatial arrangement in your clinic; your clinic includes waiting rooms, doctor’s office, staff room, etc.)**

| 6. Do not know | 5.Very satisfied | 4. Satisfied | 3. Neither satisfied not dissatisfied | 2. Dissatisfied | 1. Very dissatisfied |
| --- | --- | --- | --- | --- | --- |

3. Is the **clinic building:**

| 4. Other | 3. New/ Modern  (+1 point: the clinic's building) | 1. Decent/ Standard   (+1 point: the clinic's building) | 1. Old /Old-fashioned |
| --- | --- | --- | --- |

4. Does the **clinic** include:

| Other | Yes | No |  |
| --- | --- | --- | --- |
|  | (+1 point: the clinic's building) |  | (1) Free parking in the area? |
|  | (+1 point: the clinic's building) |  | (2) Designated parking for physicians |
|  | (+1 point: the clinic) |  | (3) Meeting room for staff meetings |
|  | (+1 point: the clinic) |  | (4) Designated staff bathroom |
|  | (+1 point: the clinic) |  | (5) Clean and convenient bathroom |

5. Are there background noises (from a busy street / nearby construction site, etc.)?

| 5. Other | 4. Very loud background noise | 3. Loud background noise | 2. Some background noise  (+1 point: the clinic) | 1. There is no noise  (+1 point: the clinic) |
| --- | --- | --- | --- | --- |

**The clinic’s waiting room**

6.Is there a window in the **waiting room?**

| 4. Other | 3. There is a window that can be opened  (+1 point: the waiting room) | 2. There is a window that cannot be opened | 1. There is no window |
| --- | --- | --- | --- |

7. **Waiting room** furniture is

| 4. Other | 3. New/ Modern (+1 point: the waiting room) | 2. Decent/ Standard  (+1 point: the waiting room) | 1. Old /Old-fashioned |
| --- | --- | --- | --- |

8. The lighting in the **waiting room** consists of

| 3. Other | 2. Yellow light | 1. White light  (+1 point: the waiting room) |
| --- | --- | --- |

8. The **waiting room** contains

| Other | Yes | No |  |
| --- | --- | --- | --- |
|  | (+1 point: the waiting room) |  | (1) Queueing system / QFLOW |
|  | (+1 point: the waiting room) |  | (2) Magazines |
|  | (+1 point: the waiting room) |  | (3) Medical information |
|  | (+1 point: the waiting room) |  | (4) Games/toys for children |
|  | (+1 point: the waiting room) |  | (5) Television |
|  | (+1 point: the waiting room) |  | (6) Music |

**Doctor’s office**

9. The door of the **doctor’s office**

| 3. Other | 2. Can be opened from outside the room using a key/card/etc. | 1. Can be opened freely from outside the room (+1 point: the doctor's office) |
| --- | --- | --- |

10. The lighting in the **doctor’s office** consists of

| 3. Other | 2. Yellow light | 1. White light  (+1 point: the doctor's office) |
| --- | --- | --- |

11. Is there a window in the **doctor’s office?**

| 4. Other | 3. There is a window that can be opened  (+1 point: the doctor's office) | 2. There is a window that cannot be opened | 1. There is no window |
| --- | --- | --- | --- |

12. The **doctor’s office** furniture is

| 4. Other | 3. New/ Modern (+1 point: the doctor's office) | 2. Decent/ Standard (+1 point: the doctor's office) | 1. Old /Old-fashioned |
| --- | --- | --- | --- |

13**.** The **doctor’s room** contains

| Other | Yes | No |  |
| --- | --- | --- | --- |
|  | (+1 point: the doctor's office) |  | (1) Adjustable chair |
|  | (+1 point: the doctor's office) |  | (2) Footrest |
|  | (+1 point: the doctor's office) |  | (3) Pictures on the walls |
|  | (+1 point: the doctor's office) |  | (4) Closet |
|  | (+1 point: the doctor's office) |  | (5) Examination bed |
|  | (+1 point: the doctor's office) |  | (6) Clean workspace |
|  | (+1 point: the doctor's office) |  | (7) Music |

14. **Staff room (if your clinic does not have a staff room, please mark “No” for all the clauses of this question)**

| Other | Yes | No |  |
| --- | --- | --- | --- |
|  | (+1 point: the staff room) |  | (1) Is there a staff room? |
|  | (+1 point: the staff room) |  | (2) Is the staff room on the same floor as the doctor’s office? |
|  | (+1 point: the staff room) |  | (3) Are there cups/milk/coffee in the staff room? |
|  | (+1 point: the staff room) |  | (4) A coffee machine |
|  | (+1 point: the staff room) |  | (5) Microwave |
|  | (+1 point: the staff room) |  | (6) Window |

**15. The following sentences describe non-physical aspects of the work environment, such as workload and working conditions. To what extent do the following sentences relate to your work environment as you experience it. Please note, there are no right or wrong answers, it is your experience that counts.**

| 7 | 6 | 5 | 4 | 3 | 2 | 1 |  |
| --- | --- | --- | --- | --- | --- | --- | --- |
| **Almost always** | **Very often** | **Often** | **Sometimes** | **Rarely** | **Very rarely** | **Almost never** |  |
| 7 | 6 | 5 | 4 | 3 | 2 | 1 | I have many patients, a heavy workload and too little time to address them |
| 7 | 6 | 5 | 4 | 3 | 2 | 1 | I have too many administrative assignments and too little time to complete them |
| 7 | 6 | 5 | 4 | 3 | 2 | 1 | My organization lacks emotional support related to illness, death, and personal difficulties of patients |
| 7 | 6 | 5 | 4 | 3 | 2 | 1 | I can influence, make decisions and initiate |
| 7 | 6 | 5 | 4 | 3 | 2 | 1 | My work is diverse and interesting |
| 7 | 6 | 5 | 4 | 3 | 2 | 1 | The salary and conditions I am given are fair in comparison to those in other workplaces |
| 7 | 6 | 5 | 4 | 3 | 2 | 1 | My work makes it hard for me to combine work and home needs |
| 7 | 6 | 5 | 4 | 3 | 2 | 1 | I feel protected against physical and verbal violence in my workplace |
| 7 | 6 | 5 | 4 | 3 | 2 | 1 | There is an atmosphere of cooperation and support among the workers in my team |
| 7 | 6 | 5 | 4 | 3 | 2 | 1 | I believe tensions between workers of different positions in the organization negatively affect my work |

**16. Following are questions regarding your feelings about your work. Following are several sentences describing feelings all workers might experience in their work. Please answer how often you have felt this way in the past 30 workdays.**

| 7 | 6 | 5 | 4 | 3 | 2 | 1 |  |
| --- | --- | --- | --- | --- | --- | --- | --- |
| **Almost always** | **Very often** | **Often** | **Sometimes** | **Rarely** | **Very rarely** | **Almost never** |  |
|  |  |  |  |  |  |  | 1. I feel tired |
|  |  |  |  |  |  |  | 1. I have no energy for going to work in the morning |
| 7 | 6 | 5 | 4 | 3 | 2 | 1 | 1. I feel physically drained. |
| 7 | 6 | 5 | 4 | 3 | 2 | 1 | 1. I feel fed up. |
| 7 | 6 | 5 | 4 | 3 | 2 | 1 | 1. I feel like my “batteries” are “dead”. |
| 7 | 6 | 5 | 4 | 3 | 2 | 1 | 1. I feel burnt out. |
|  |  |  |  |  |  |  | 1. My thinking process is slow. |
| 7 | 6 | 5 | 4 | 3 | 2 | 1 | 1. I have difficulties concentrating. |
| 7 | 6 | 5 | 4 | 3 | 2 | 1 | 1. I feel I am not thinking clearly. |
|  |  |  |  |  |  |  | 1. I feel I'm not focused in my thinking |
| 7 | 6 | 5 | 4 | 3 | 2 | 1 | 1. I have difficulty thinking about complex things. |
|  |  |  |  |  |  |  | 1. I feel I am unable to be sensitive to the needs of patients or coworkers. |
| 7 | 6 | 5 | 4 | 3 | 2 | 1 | 1. I feel I am not capable of investing emotionally in my patients or coworkers. |
| 7 | 6 | 5 | 4 | 3 | 2 | 1 | 1. I feel I am not capable of being sympathetic to my patients or coworkers. |

**General background questions**

The following questions are used for statistical analysis and will allow us to adjust the survey’s result to different populations of doctors.

**17. Employment background**

| Possible answers | Question |
| --- | --- |
|  | How long have you been practicing your profession (in years) |
| GP  Family medicine specialist  Family medicine resident  Internal medicine specialist  Other specialty  Other | What is your medical specialty |
| One  Two or more | How many clinics do you work in? |
|  | How many hours do you spend in your primary clinic in a week? |
| HMO clinic  Independent clinic  Branch  A compound with multiple clinics (Beit Rof’im)  Other | Is your primary workplace a |
| 0-3 months  3-12 months  1-5 years  5-10 years  Over 10 years | How long have you been working in your primary workplace (clinic) |
|  | What is your age? |
|  | What is your gender? |

| 7 | 6 | 5 | 4 | 3 | 2 | 1 |  |
| --- | --- | --- | --- | --- | --- | --- | --- |
| **Almost always** | **Very often** | **Often** | **Sometimes** | **Rarely** | **Very rarely** | **Almost never** |  |
| 7 | 6 | 5 | 4 | 3 | 2 | 1 | The following question concerns financial hardship – to what extent do you feel your financial state is strained. For example, having to borrow money to pay bills or cut back on essential expense such as clothing or groceries |

**Thank you!**
